# Intraoperative detection of a right atrial mass during holmium: YAG laser lithotripsy: A fatal and rare complication: A case report

**DOI:** 10.1097/MD.0000000000046530

**Published:** 2025-12-12

**Authors:** Yuwei Zhou, Ruifen Li

**Affiliations:** a Department of Ultrasound, Tangshan People’s Hospital, Tangshan City, Hebei Province, China.

**Keywords:** Holmium:YAG laser, intraoperative echocardiography, obstructive shock, right atrial mass, ureteral stone

## Abstract

**Rationale:**

Holmium:YAG laser lithotripsy is widely used for the treatment of urinary tract stones and is generally considered safe. However, cardiovascular complications such as intracardiac masses are exceedingly rare and potentially fatal. This report presents a case in which an unexpected right atrial mass was detected intraoperatively during laser lithotripsy under general anesthesia using real-time transthoracic echocardiography.

**Patient concerns:**

A 54-year-old woman with right-sided hydronephrosis and ureteral calculi was admitted for elective holmium:YAG laser lithotripsy. Preoperative evaluations revealed elevated urinary leukocytes and C-reactive protein, with no evidence of cardiovascular abnormalities.

**Diagnoses:**

During the procedure, the patient developed sudden hypotension, tachycardia, and hypoxia. Emergency bedside transthoracic echocardiography revealed a large hypoechoic mass in the right atrium obstructing blood flow. Although the exact nature of the mass could not be confirmed, findings were highly suggestive of an acute thromboembolic process.

**Interventions:**

Immediate thrombolytic therapy with urokinase was administered, accompanied by continuous ultrasound monitoring. The mass showed evidence of fragmentation and partial resolution. Despite transient improvement in hemodynamics, the patient experienced cardiac arrest several hours later and could not be resuscitated.

**Outcomes:**

No autopsy was performed. Based on clinical deterioration, imaging progression, and absence of other identifiable causes, a fatal cardiac event, potentially related to embolization of an intracardiac mass, was considered the most probable cause of death.

**Lessons:**

This case highlights the importance of preoperative and intraoperative cardiovascular monitoring in urologic patients, particularly those at risk for infection-related hypercoagulability. Bedside transthoracic echocardiography facilitates the early detection of newly developed or evolving acute intracardiac events, and in future cases, pulsed-wave tissue Doppler imaging may be considered to further assess thrombus mobility and embolic risk. For future high-risk patients, preoperative evaluation, including thromboelastography or other laboratory tests, and timely or prophylactic antibiotic administration may help identify occult hypercoagulability and reduce infection-related thrombotic risk. Postoperatively, low-dose heparin can be cautiously considered under close laboratory supervision to manage thrombotic risk.

## 1. Introduction

Holmium:YAG laser lithotripsy is a standard endoscopic procedure for the management of urinary tract calculi, owing to its minimally invasive nature and high efficacy.^[1]^ Although generally regarded as safe, rare systemic complications may occur during the perioperative period.^[2]^ Cardiovascular adverse events during endourological interventions are uncommon,^[3]^ and the intraoperative occurrence of intracardiac masses, particularly right atrial masses or embolic events, remains exceedingly rare, with existing literature limited to isolated case reports.^[4]^

The rapid development of a right atrial mass poses a critical threat to hemodynamic stability, potentially leading to cardiac arrest or pulmonary artery obstruction, thereby carrying a high risk of mortality.^[5]^ Potential etiologies include infection, coagulation disorders, central venous catheter-related reactions, or endothelial activation induced by bacterial toxins.^[6–8]^ Such complications often present abruptly and nonspecifically, making timely diagnosis particularly challenging.

Imaging techniques play a critical role in the assessment of sudden intracardiac abnormalities occurring intraoperatively. In particular, bedside echocardiography enables real-time visualization of cardiac structures and hemodynamics, facilitating precise localization, morphological characterization, and dynamic monitoring of intracardiac masses, thereby informing clinical decision-making.^[9]^

Herein, we report a rare and fatal cardiovascular complication occurring during Holmium:YAG laser lithotripsy. The patient had no evident cardiovascular symptoms preoperatively but had a history of urinary tract infection. Intraoperative echocardiography revealed a newly developed intracardiac echogenic mass in the right atrium. Despite prompt intervention, the patient succumbed to postoperative pulmonary embolism-related complications. This report presents a detailed case analysis and discusses relevant literature to enhance awareness of this underrecognized complication.

## 2. Case report

**Figure 1.**
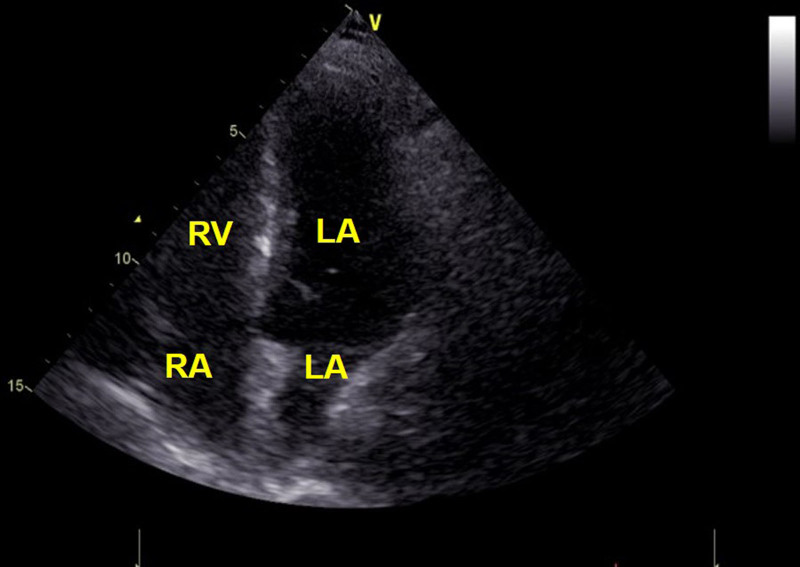
Preoperative transthoracic echocardiography (TTE) in the apical 4-chamber view demonstrates normal morphology of the right atrium and right ventricle, with no evidence of thrombus or intracardiac mass. LA = left atrium, LV = left ventricle, RA = right atrium, RV = right ventricle.

A 54-year-old woman presented with right lower abdominal pain persisting for more than 10 days, which had worsened over the past day. She had no history of hypertension, diabetes mellitus, or coronary artery disease. The patient had not received any anticoagulant, antiplatelet, or hormonal medications prior to admission. Physical examination revealed right renal tenderness and costovertebral angle percussion pain. Initial laboratory tests showed leukocytosis, and urinalysis indicated microscopic hematuria. Preoperative coagulation profile demonstrated a prothrombin time of 14 seconds (slightly prolonged) and an elevated D-dimer level of 717 ng/mL, while other coagulation parameters and platelet count were within normal limits, indicating no significant baseline coagulopathy but a potential thrombotic tendency. The patient denied any personal or family history of bleeding disorders, thrombophilia, or thromboembolic events. Electrocardiography was unremarkable. Transthoracic echocardiography (TTE) revealed no other structural abnormalities (Fig. 1). Abdominal ultrasonography and computed tomography (CT) revealed multiple calculi in the right kidney and a 1.2 cm calculus in the distal right ureter, accompanied by dilation of the renal pelvis and ureter. The patient was scheduled for transurethral holmium: YAG laser lithotripsy under general anesthesia.

**Figure 2.**
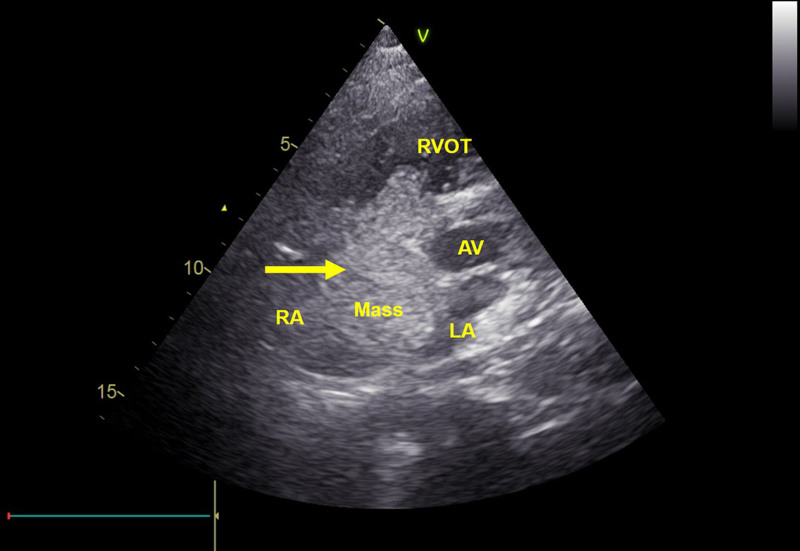
Bedside TTE in a nonstandard short-axis view of the great arteries reveals a large hypoechoic mass in the right ventricular inflow tract, attached to the atrial side of the septal tricuspid leaflet. LA = left atrium, RA = right atrium, RVOT = right ventricular outflow tract, AV = aortic valve.

Anesthesia induction and endotracheal intubation were uneventful. Approximately 15 minutes into the procedure, the patient developed sudden hypoxemia with oxygen saturation dropping to 75%, hypotension (blood pressure 52/27 mm Hg), and tachycardia (heart rate 146 bpm). The surgery was immediately suspended. Bedside TTE revealed a newly appeared, mobile, hypoechoic mass in the right atrium, measuring approximately 36 × 23 mm (Fig. 2). The mass was attached to the septal leaflet of the tricuspid valve, prolapsing into the right ventricle during diastole and returning to the atrium during systole, resulting in significant obstruction of right-sided inflow. This mass had not been observed on the preoperative TTE. The first postoperative coagulation test demonstrated markedly elevated D-dimer levels (>69,000 ng/mL) and fibrin degradation products (FDP, 189.56 μg/mL), indicating extensive activation of the coagulation and fibrinolytic systems.

An emergent multidisciplinary consultation was convened including cardiology, anesthesiology, urology, and critical care. Although the exact nature of the intracardiac mass (thrombus vs non-thrombotic embolus) remained undetermined, the presence of acute hypoxemia, hemodynamic instability, right heart dilation, and echocardiographic features of a free-floating mass raised strong suspicion for a life-threatening pulmonary embolism or intracardiac obstruction. Given the patient’s critical clinical condition and evidence of significant right ventricular strain, immediate intravenous thrombolytic therapy with urokinase was initiated as a life-saving measure.

**Figure 3.**
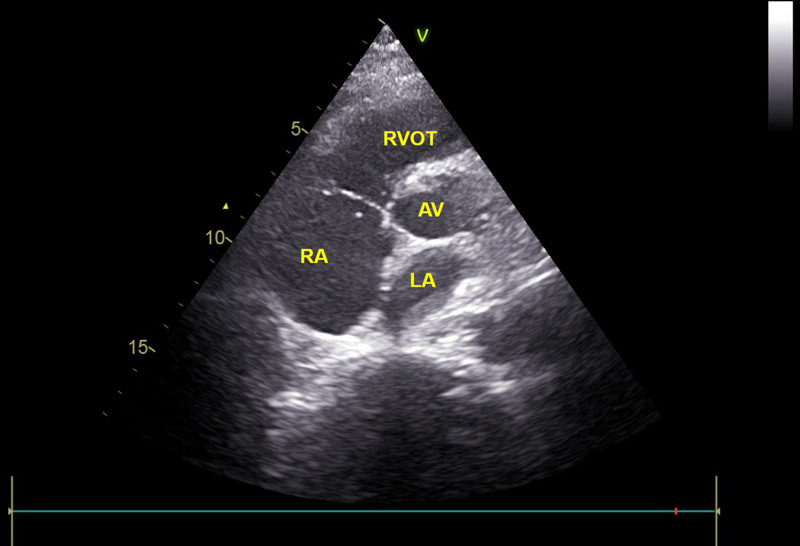
Post-thrombolysis beside TTE in a nonstandard short-axis view of the great arteries shows complete resolution of the intracardiac mass 1 hour after intravenous thrombolytic therapy. LA = left atrium, RA = right atrium, RVOT = right ventricular outflow tract, AV = aortic valve.

Follow-up TTE one hour after thrombolysis showed complete resolution of the right atrial mass and improvement in right heart structure and function (Fig. 3). The patient was subsequently transferred to the intensive care unit for continued monitoring. Following cardiothoracic consultation, unfractionated heparin was continued postoperatively, but was promptly discontinued 3 hours after intensive care unit admission due to critical coagulation abnormalities (activated partial thromboplastin time [aPTT], 215.7 seconds; prothrombin time [PT], 67.6 seconds), reflecting effective anticoagulation while substantially increasing bleeding risk. The patient remained under intensive monitoring. Approximately 5 hours later, the patient experienced sudden cardiovascular collapse characterized by bradycardia (heart rate 41 bpm), hypotension (blood pressure 46/23 mm Hg), and undetectable oxygen saturation and pulse. Bedside TTE during the episode showed severe right-heart enlargement, reduced transvalvular flow, and low-amplitude contractions, without residual or recurrent thrombus. Despite aggressive resuscitative efforts, the patient was pronounced clinically deceased.

## 3. Discussion

In recent years, the incidence of ureteral calculi has shown a steady upward trend.^[10]^ For distal ureteral calculi, transurethral ureteroscopic holmium laser lithotripsy has become the preferred first-line therapeutic option. Due to its high fragmentation efficiency, wide applicability across various stone compositions, minimal damage to the ureteral wall, and high stone clearance rates, holmium laser lithotripsy has gained widespread clinical acceptance. Its safety has been well documented in numerous case series.^[11,12]^ While most intraoperative complications involve the urinary tract, such as iatrogenic trauma or infection, serious cardiovascular events remain exceedingly rare.^[13]^

In this case, the patient developed acute intraoperative hemodynamic deterioration. Bedside transthoracic echocardiography identified a newly emerged hypoechoic mass in the right atrium, accompanied by right heart overload and interventricular septal motion abnormalities. In the absence of preexisting cardiovascular disease and with unremarkable preoperative imaging findings, alternative diagnoses such as tumor, hematoma, or foreign body were effectively excluded. However, the family’s refusal to permit autopsy precluded definitive histopathological confirmation. Nevertheless, the sudden onset and rapid progression of the intracardiac mass were highly suggestive of intraoperative thrombus formation.

Right atrial thrombus is an exceptionally rare clinical condition, primarily attributed to the distinct anatomical and physiological characteristics of the right atrium.^[14]^ As the chamber responsible for receiving venous return from both the superior and inferior vena cava, the right atrium exhibits high-velocity and turbulent blood flow, which serves as a natural deterrent to thrombus formation. In addition, its smooth endocardial surface and relatively simple architecture, lacking valves or major flow-disturbing structures, further minimize thrombogenic risk.^[15]^ Most right atrial thrombus described in the literature have been detected incidentally, either during postoperative imaging or at autopsy,^[16]^ and frequently occur in patients without an identifiable hypercoagulable condition. Similarly, our patient had no history of cardiovascular disease, central venous catheter placement, or hematologic abnormalities, and exhibited no clinical signs suggestive of hypercoagulability. However, her prolonged history of urinary tract calculi and marked preoperative pyuria may have indicated a subclinical infection that potentially contributed to a prothrombotic state.

From a pathophysiological perspective, multiple intraoperative factors may have acted synergistically to precipitate acute thrombus formation, in accordance with Virchow’s triad: hemodynamic alterations, endothelial injury, and a hypercoagulable state.^[17]^ General anesthesia can reduce venous return, while the lithotomy position may exacerbate venous stasis in the lower extremities.^[18,19]^ In addition, urethral catheterization and holmium laser lithotripsy may induce microtrauma to the urogenital mucosa or adjacent vascular structures.^[20]^ Intraoperative physiological stress, potential infection, and a preexisting mild hypercoagulable state may collectively trigger the coagulation cascade, resulting in a transient hypercoagulable state and ultimately promoting thrombus formation within the right atrium.^[21]^

Regarding the origin of the thrombus, 2 primary mechanisms were considered: in situ formation within the right atrium secondary to intraoperative hypercoagulability, and embolization of a peripheral deep vein thrombus via the inferior vena cava. However, in this case, preoperative duplex ultrasonography of both lower extremities revealed no evidence of deep vein thrombosis, and the patient had no prior history of central venous catheter placement or venous thromboembolism, thereby making an embolic source less likely.

Intraoperative transthoracic echocardiography revealed a mobile, floating mass within the right atrium, which may have intermittently obstructed the tricuspid valve orifice. This likely resulted in impaired right ventricular filling, reduced cardiac output, and subsequent cerebral hypoperfusion. Following the administration of urokinase, the mass temporarily disappeared on imaging, accompanied by a transient improvement in hemodynamic status. However, the patient soon experienced cardiac arrest. Based on the clinical course and dynamic echocardiographic findings, embolization of the thrombus into the pulmonary artery was suspected, leading to acute pulmonary embolism and obstructive shock. In such scenarios, abrupt occlusion of the pulmonary artery can result in sudden right ventricular dilation, elevated pulmonary arterial pressures, diminished cardiac output, and ultimately cardiogenic shock with cerebral hypoperfusion, culminating in irreversible brain injury and multi-organ failure. Although sinus rhythm was briefly restored following cardiopulmonary resuscitation, the patient rapidly progressed to brainstem areflexia and profound circulatory collapse. The immediate cause of death was therefore considered refractory cardiogenic and obstructive shock secondary to acute right atrial thrombus with pulmonary embolism and subsequent multiorgan failure. This case highlights the fulminant progression and exceedingly narrow therapeutic window associated with such events.

Notably, although rare cases of pulmonary embolism caused by retrograde migration of stone fragments into the venous system have been reported following pneumatic lithotripsy,^[4]^ the present patient did not undergo this procedure. The intracardiac mass was identified intraoperatively via ultrasound, and its echogenic characteristics were inconsistent with those of urinary calculi. Moreover, the patient had no history of central venous catheter placement. These findings collectively support the diagnosis of acute intraoperative thrombus formation and underscore the rarity and clinical distinctiveness of this complication.

In summary, this case highlights a potentially underrecognized acute intracardiac complication associated with urological endoscopic surgery. Despite the patient’s low-risk preoperative profile, the rapid development of a right atrial thrombus led to fatal circulatory collapse, underscoring the critical need for heightened vigilance toward such catastrophic cardiovascular events during these procedures. For patients undergoing high-risk surgeries, intensified intraoperative hemodynamic monitoring,^[15,22,23]^ optimized patient positioning, and thorough evaluation of hypercoagulable states are essential preventive strategies. Moreover, bedside intraoperative echocardiography can facilitate early detection of intracardiac abnormalities or flow disturbances, enabling timely intervention that may mitigate severe outcomes.

## 4. Conclusion

This case demonstrates that acute right atrial thrombus formation and fatal sequelae may occur during urological endoscopic procedures, even in patients without evident high-risk factors. Intraoperative bedside echocardiography serves as a valuable tool for early recognition, providing crucial diagnostic information and critical opportunities for prompt management.

## Author contributions

**Conceptualization:** Yuwei Zhou.

**Writing – original draft:** Yuwei Zhou.

**Writing – review & editing:** Yuwei Zhou, Ruifen Li.
